# Transmission and Antibiotic Resistance of *Achromobacter* in Cystic Fibrosis

**DOI:** 10.1128/JCM.02911-20

**Published:** 2021-03-19

**Authors:** Migle Gabrielaite, Jennifer A. Bartell, Niels Nørskov-Lauritsen, Tacjana Pressler, Finn C. Nielsen, Helle K. Johansen, Rasmus L. Marvig

**Affiliations:** a Center for Genomic Medicine, Rigshospitalet, Copenhagen, Denmark; b Department of Clinical Microbiology, Rigshospitalet, Copenhagen, Denmark; c Department of Clinical Microbiology, Aarhus University Hospital, Aarhus, Denmark; d Cystic Fibrosis Center, Rigshospitalet, Copenhagen, Denmark; e Department of Clinical Medicine, Faculty of Health and Medical Sciences, University of Copenhagen, Copenhagen, Denmark; Medical College of Wisconsin

**Keywords:** *Achromobacter*, cystic fibrosis airway infection, pathogen transmission, genomics, phylogenetics, antibiotic resistance, genetic epidemiology

## Abstract

*Achromobacter* species are increasingly being detected in patients with cystic fibrosis (CF), and this emerging pathogen is associated with antibiotic resistance and more-severe disease outcomes. Nonetheless, little is known about the extent of transmission and antibiotic resistance development in *Achromobacter* infections. We sequenced the genomes of 101 *Achromobacter* clinical isolates (identified as Achromobacter xylosoxidans based on matrix-assister laser desorption ionization–time of flight [MALDI-TOF] or API N20 typing) collected from 51 patients with CF—the largest longitudinal data set to date. We performed phylogenetic analysis on the genomes and combined this with epidemiological and antibiotic resistance data to identify patient-to-patient transmission and the development of antibiotic resistance. We confirmed that the MALDI-TOF or API N20 method was not sufficient for *Achromobacter* species-level typing and that the population of *Achromobacter* isolates was composed of five different species, among which A. xylosoxidans accounted for 52% of infections. Most patients were infected by unique *Achromobacter* clone types; nonetheless, suspected patient-to-patient transmission cases identified by shared clone types were observed in 35% (*n* = 18) of patients. In 15 of 16 cases, the suspected transmissions were further supported by genome- or clinic visit-based epidemiological analysis. Finally, we found that resistance developed over time. We show that whole-genome sequencing (WGS) is essential for *Achromobacter* species typing and identification of patient-to-patient transmission, which was revealed for Achromobacter ruhlandii, A. xylosoxidans, and, for the first time, Achromobacter insuavis. Furthermore, we show that the development of antibiotic resistance is associated with chronic *Achromobacter* infections. Our findings emphasize that transmission and antibiotic resistance should be considered in future treatment strategies.

## Abstract (precis)

*Achromobacter* species are increasingly being detected in patients with cystic fibrosis (CF), and this emerging pathogen is associated with antibiotic resistance and more-severe disease outcomes. Nonetheless, little is known about the extent of transmission and antibiotic resistance development in *Achromobacter* infections.

## INTRODUCTION

The majority of patients with cystic fibrosis (CF) are affected by bacterial airway infections which persist for years and often are the cause of respiratory failure and premature death (1). Pseudomonas aeruginosa remains the most common pathogen causing infections in patients with CF airways (1, 2); however, *Achromobacter* is an emerging and less-studied opportunistic pathogen (3, 4). Understanding of bacterial antibiotic resistance development and transmission is crucial for effective pathogen management and elimination (5–8). For example, the Achromobacter ruhlandii Danish epidemic strain (DES) has already been defined as a hypermutable and antibiotic-resistant clone type that has been transmitted among Danish patients with CF (9–11).

Here, we sequenced and analyzed the genomes of the largest collection of *Achromobacter* clinical isolates from patients with CF to date. First, we aimed to assess how species-level *Achromobacter* typing based on whole-genome sequencing (WGS) compares to species typing based on biochemical or mass spectrometry methods, which were used for routine clinical diagnostics. Second, we aimed to use genetic distance and the phylogenetic relationship of genomes to identify cases of *Achromobacter* transmission between patients, and also to include other epidemiological data in order to identify possible drivers of transmission. Third, we aimed to investigate and present the extent of antibiotic resistance development in the light of genetic epidemiological findings. Overall, we aimed to better understand patient-to-patient transmission and the development of antibiotic resistance in *Achromobacter* during infections in patients with CF, ultimately leading to improved strategies for handling persistent airway infections.

## MATERIALS AND METHODS

### Bacterial isolates.

The analysis included 101 *Achromobacter* clinical isolates that, prior to this study, were identified in the routine clinical microbiology laboratory as Achromobacter xylosoxidans by API N20 (bioMérieux, France) or matrix-assisted laser desorption ionization–time of flight (MALDI-TOF) (Bruker, Germany) typing. The isolates were sampled from 51 patients with CF attending the Copenhagen Cystic Fibrosis Center at Rigshospitalet, Denmark. This data set represents 49% (51 of 104) of all patients attending the Copenhagen Cystic Fibrosis Center with A. xylosoxidans detected at least once (as defined by MALDI-TOF or API N20 typing) in the years 2002 to 2018 (detailed descriptions of patients are provided in Table S1 in the supplemental material). We included isolates sampled before 2002 for nine of the patients; however, samples from patients with *Achromobacter* detected only prior to 2002 were not included in the study. Four isolates had been analyzed by Veschetti et al. in 2020 (12); patients A and B in their report correspond to patients P0802 and P8603, respectively, in this study. The use of clinical isolates was approved by the local ethics committee at the Capital Region of Denmark (Region Hovedstaden; approval registration number H-4-2015-FSP), and the use of clinical registry data was approved by the Danish Agency for Patient Safety (approval registration number 31-1521-428).

### Antibiotic treatment.

All patients received early antibiotic treatment for *Achromobacter* at the first positive culture. All treatments were based on antibiotic susceptibility testing. The most frequently used treatment regimen was inhalations of colistin (CST) in combination with amoxicillin-clavulanic acid (AMC) for 3 weeks (4). If early eradication treatment failed, other treatment modalities were used: mainly 14 days of intravenous treatment with either piperacillin-tazobactam (TZP) or meropenem (MEM), or ceftazidime (CAZ) in combination with tobramycin (TOB) and trimethoprim-sulfamethoxazole (SXT). In some cases, patients were treated with inhaled or orally administered colistin or ceftazidime.

### Bacterial genome sequencing and definition of clone type.

Genomic DNA was extracted and purified from *Achromobacter* clones with the DNeasy blood and tissue kit (Qiagen). Genomic DNA libraries were prepared using a Nextera XT DNA Library Prep kit (Illumina), and libraries were sequenced on an Illumina MiSeq instrument generating 250-base paired-end sequencing reads (average, 1,124,551 read pairs; range, 350,677 to 2,118,817 read pairs). Clone types were defined by Pactyper (13) using the default settings and a species core genome defined by GenAPI using the default parameters and the 101 *Achromobacter* isolates from this study (14). Multilocus sequence typing (MLST) sequence types (STs) were assigned using the *de novo* assembly-based mlst tool (15) and the PubMLST database with an *Achromobacter* sp. scheme (16).

### *De novo* assembly-based phylogenetic tree generation.

Sequence reads from each isolate were corrected and assembled into scaffolds by SPAdes, version 3.10.1 (17), using default settings and k-mer sizes ranging from 21 to 127 bases. Genome assemblies consisted, on average, of 216 scaffolded contigs (range, 92 to 506). Core genome single-nucleotide-variant (SNV)-based phylogenetic trees of the 101 *de novo*-assembled *Achromobacter* isolates together with publicly available reference genomes were generated with parsnp, version 1.2 (18), using default settings. Eight complete *Achromobacter* reference genomes were included in the phylogenetic analysis (RefSeq assembly accession numbers GCF_000165835.1 [Achromobacter aegrifaciens], GCF_000758265.1 [A. xylosoxidans], GCF_001051055.1 [A. ruhlandii], GCF_001457475.1 [A. xylosoxidans], GCF_001558755.2 [Achromobacter insuavis], GCF_001558915.1 [A. ruhlandii], GCF_001559195.1 [A. xylosoxidans], and GCF_900475575.1 [A. xylosoxidans]). The phylogenetic tree was visualized with the Microreact Web server (19). The phylogenetic tree of A. ruhlandii clone type AX01DK01 isolates together with 19 A. ruhlandii DES genomes from the study of Ridderberg et al. (2020) (10) was based on core genome SNVs using parsnp, version 1.2 (18), with the default settings and was visualized with the iTOL Web server (20).

### Patient-to-patient transmission identification.

Phylogenies and genetic distances of patient isolates that shared a clone type and were suspected to participate in patient-to-patient transmission events were determined with BacDist (for SNV-based phylogenetic relationships and pairwise SNV distances) (21) and GenAPI (for gene content differences) (14). For alignments to the reference genome, GCF_001051055.1 was used for A. ruhlandii, GCF_001558755.2 for A. insuavis, and GCF_001457475.1 for A. xylosoxidans isolates. All the reference genomes used had average nucleotide identities of ≥95% to our isolates as identified by fastANI (22). BacDist, which was developed in-house and has been used in other microbial genomics studies (23, 24), is a Snakemake workflow engine (25) that first performs variant calling with Snippy, verison 4 (26), using a minimum read mapping quality of 50, a minimum read coverage of 10, and a minimum fraction of reads supporting the variant of 0.5 for each sample from the sample group submitted. Then the variant calls shared by all isolates (>80% of reads supporting the variant) are filtered out in order to keep only variants introduced during the infection, i.e., variants that differentiate isolates of the same lineage. Furthermore, poorly covered (<10-fold read coverage) positions are excluded from the final variant list and all calculations. We also used an optional feature to identify the sites of possible recombination with ClonalFrameML (27). Possible recombination events were rare and affected minor parts of the genome; therefore, they were not excluded from the overall analysis. Phylogenetic trees were generated with RAxML (28) using default parameters and the general time-reversible (GTR) CAT model. GenAPI was run with default settings that require 98% gene identity with 25% gene coverage or 90% gene identity with 50% gene coverage. Furthermore, as recommended in the GenAPI documentation, genes shorter than 150 bp were not included in the analysis. Phylogenies were visualized using the iTOL Web service (20). Detailed information about the quality of reads, alignments, and assemblies is provided in Table S2.

In our local CF database, we extracted days and wards at which the patients had registered microbial samples from 2002 through December 2018. The great majority of samples were taken when the patient presented at the ward; nonetheless, we note that a few of the samples were sent to the ward and registered without the patient being present. We used this information to identify days of possible contact between patients (referred to below as “contact days”), i.e., we were able to infer if patients had potentially been at the same hospital ward on the same date, not if they had actually met at the hospital. Clinic visit dates from 2 years prior to the first *Achromobacter*-positive sample for each patient recorded in the digital registry were included in the analysis (to account for undetected colonization/transmission) for all 51 patients with sequenced *Achromobacter* isolates in this study. The analysis was performed for each patient pair (1,275 possible combinations).

### Hypermutator identification.

Hypermutators were identified in clone types, where two or more isolates were available, by using BacDist (21) to call genetic variants, and then the transition-to-transversion (Ts/Tv) nucleotide substitution ratio was evaluated. If the Ts/Tv ratio was >3, the clone type was concluded to be hypermutable. Insertions, deletions, and frameshifts in the mismatch repair (MMR) system genes *mutL* and *mutS* were manually evaluated to identify which genetic changes could cause hypermutability (29).

### Antibiotic susceptibility testing and statistical analysis.

Rosco Diagnostica antibiotic-containing tablets and the corresponding zone-of-inhibition interpretive breakpoints were used for antibiotic susceptibility testing, and isolate susceptibility profiles were interpreted as “resistant,” “intermediately resistant,” or “susceptible” according to the manufacturer’s guidelines for *Achromobacter* spp., since no EUCAST or CLSI breakpoint standard is available for *Achromobacter*. Antibiotic susceptibility profiles were available for all 92 *Achromobacter* isolates sampled since 2002 (Table S3). Antibiotic susceptibility profiles were tested with AMC, ampicillin (AMP), aztreonam (ATM), CAZ, ceftriaxone (CRO), cefuroxime (CXM), chloramphenicol (CHL), ciprofloxacin (CIP), CST, imipenem (IPM), MEM, moxifloxacin (MXF), penicillin (PEN), TZP, rifampin (RIF), sulfamethizole (SMZ), tetracycline (TET), tigecycline (TGC), TOB, trimethoprim (TMP), and SXT. Statistical analysis of resistance phenotype distribution differences between early, late, and unique isolates was performed using the Wilcoxon signed-rank test with a *P* value of 0.05 as the significance threshold.

### Data availability.

*Achromobacter* whole-genome sequencing data are available at the European Nucleotide Archive under BioProject study accession number PRJEB39108.

## RESULTS

### Selection of *Achromobacter* isolates for sequencing.

**FIG 1:**
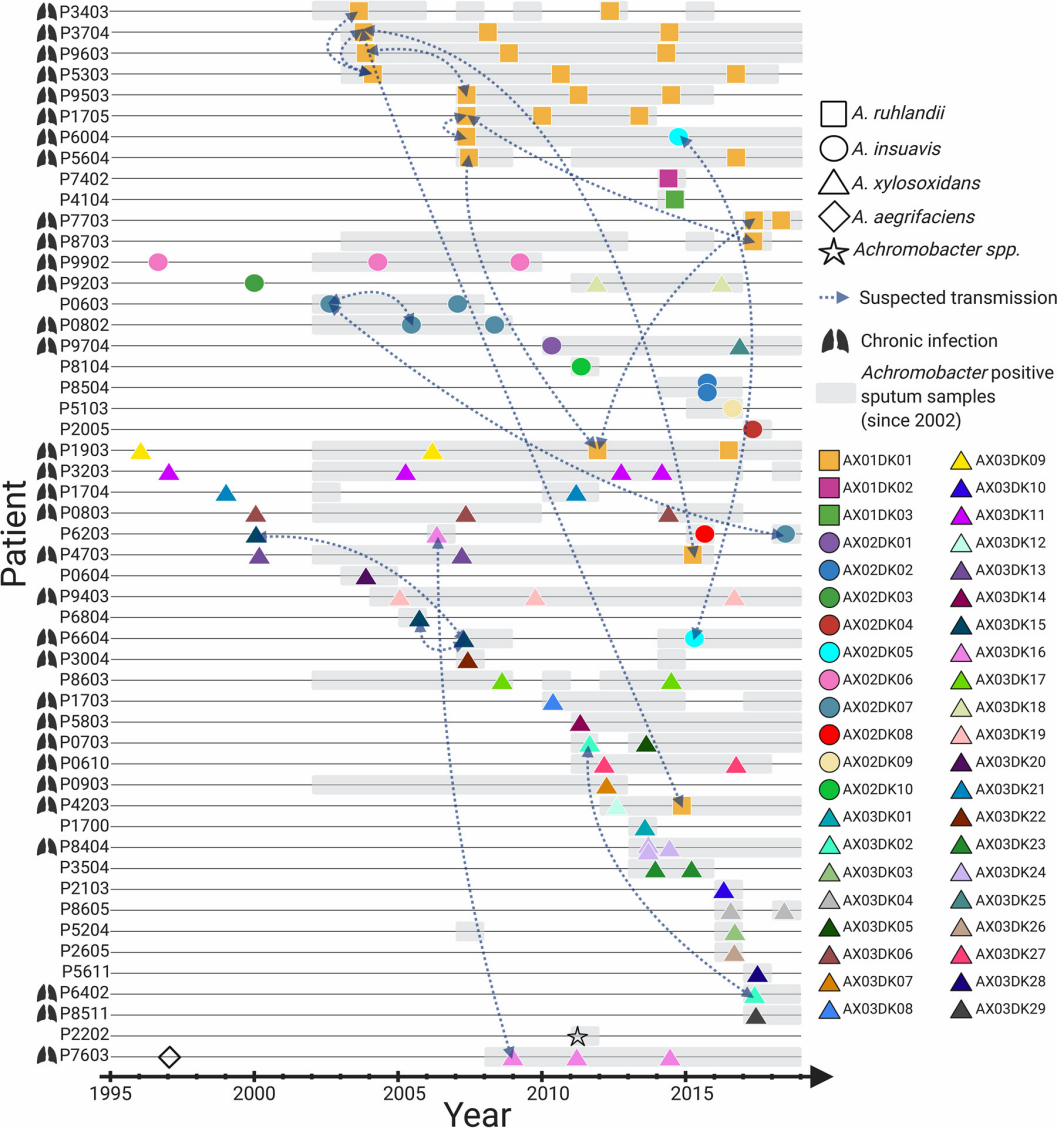
Overview of 101 longitudinally collected *Achromobacter* isolates from patients with CF.

From our local CF database, we found that 104 patients attending the Copenhagen Cystic Fibrosis Clinic in the years 2002 to 2018 had at least one culture positive for *Achromobacter* (Achromobacter xylosoxidans as defined by MALDI-TOF or API N20 typing). We sequenced the genomes of 101 *Achromobacter* isolates from 51 of the patients (Fig. 1). The isolates recovered from 2002 to 2015 were selected primarily in order to sequence the first and last isolates from patients with cultures positive for *Achromobacter* over long periods; from the year 2015 onward, all isolates from any patient were selected for genome sequencing. Also, we included isolates sampled before 2002 for nine of the patients.

For 30 patients, we sequenced at least two longitudinally collected isolates (range, 2 to 4 isolates), and for 21 patients, we sequenced only a single isolate each (*n* = 20) or two isolates from the same time point (*n* = 1; patient P8504). The time between the first and the last sequenced isolate from patients with longitudinal isolates ranged from 1 to 20 years. Patients P9503 and P9603 were siblings.

Of the 51 patients, 32 (63%) were clinically defined as chronically infected with *Achromobacter*, i.e., half or more of their samples were positive for *Achromobacter* over a year when at least 4 samples were taken, or when there were 4 or more specific precipitating antibodies against *Achromobacter* (30). Of the 53 patients for whom no isolates were included in the study, 15 (28%) were clinically defined as chronically infected with *Achromobacter* (see Table S1 in the supplemental material).

### *Achromobacter* species typing.

Prior to this study, all isolates included were identified as A. xylosoxidans species in the routine clinical microbiology laboratory by MALDI-TOF or API N20 typing.

**FIG 2:**
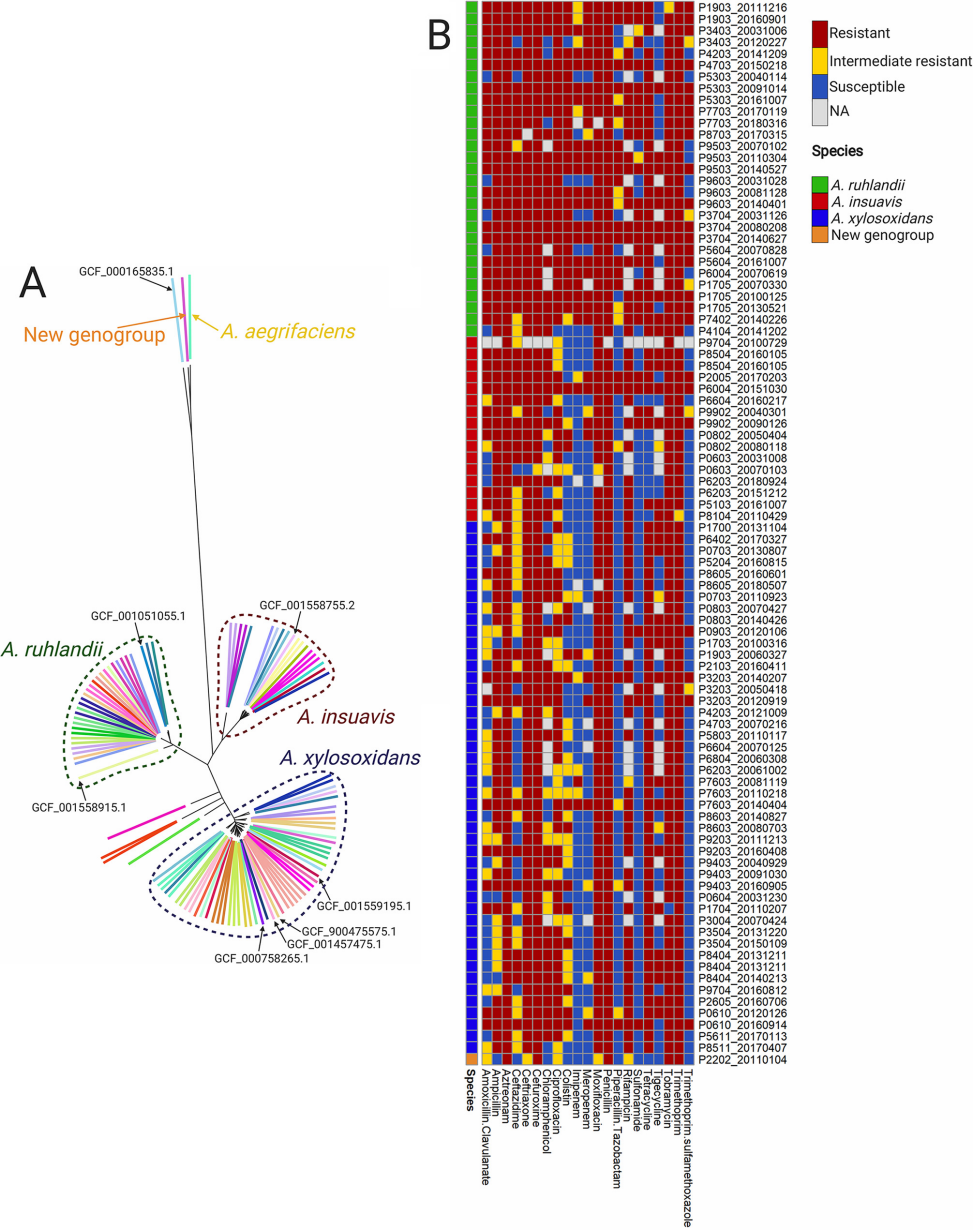
Population structure of *Achromobacter* clinical isolates and their susceptibilities to antibiotics. (A) Phylogenetic tree of 101 *Achromobacter* clinical isolates together with eight *Achromobacter* reference genomes. Colored lines represent bacterial isolates from different patients; arrows point to *Achromobacter* reference genomes. The phylogenetic tree can be accessed on the Microreact Web server (https://microreact.org/project/ByZx4dqC7). (B) Overview of susceptibility profiles of 92 *Achromobacter* isolates against 21 antibiotics.

Nonetheless, when we compared our *Achromobacter* isolate genome sequences to eight publicly available complete *Achromobacter* reference genomes, we found that our isolate collection was composed of five different *Achromobacter* species (Fig. 2A). According to our findings, 15 (25%) patients were infected with A. ruhlandii (AX01 clone types), 12 (20%) with A. insuavis (AX02), 31 (52%) with A. xylosoxidans (AX03), and 2 with other *Achromobacter* species (Achromobacter aegrifaciens and a new genogroup [AX04DK01]). Since only a single isolate each was available for A. aegrifaciens and the new genogroup, these isolates were excluded from further analysis.

### Clonal identities of isolates.

We further compared the genomes to determine the clonal identities of isolates. Isolates that differed by <5,000 SNVs in the core genome were assigned the same clone type. The minimum pairwise distances observed between isolates from different clone types were 41,237, 32,339, and 8,047 SNVs for A. ruhlandii, A. insuavis, and A. xylosoxidans, respectively, and the maximum pairwise distances observed between isolates belonging to the same clone type were 1,634, 482, and 1,160 SNVs for A. ruhlandii, A. insuavis, and A. xylosoxidans, respectively. Of the 30 patients for whom we had longitudinally collected isolates, 21 patients were infected with the same clone type over time, and 10 patients were infected with more than one clone type (*n* = 2) and/or species (*n* = 9) of *Achromobacter* (Fig. 1).

We also used a publicly available MLST scheme on *de novo* assemblies for *Achromobacter* to identify the ST of each isolate. We were able to identify STs for 60 of 101 isolates, and we found that isolates of the same clone type also had the same ST. For 25 of 44 clone types, no ST could be assigned (Table S4 presents more details on the STs).

### Patient-to-patient transmission in three *Achromobacter* species.

**FIG 3:**
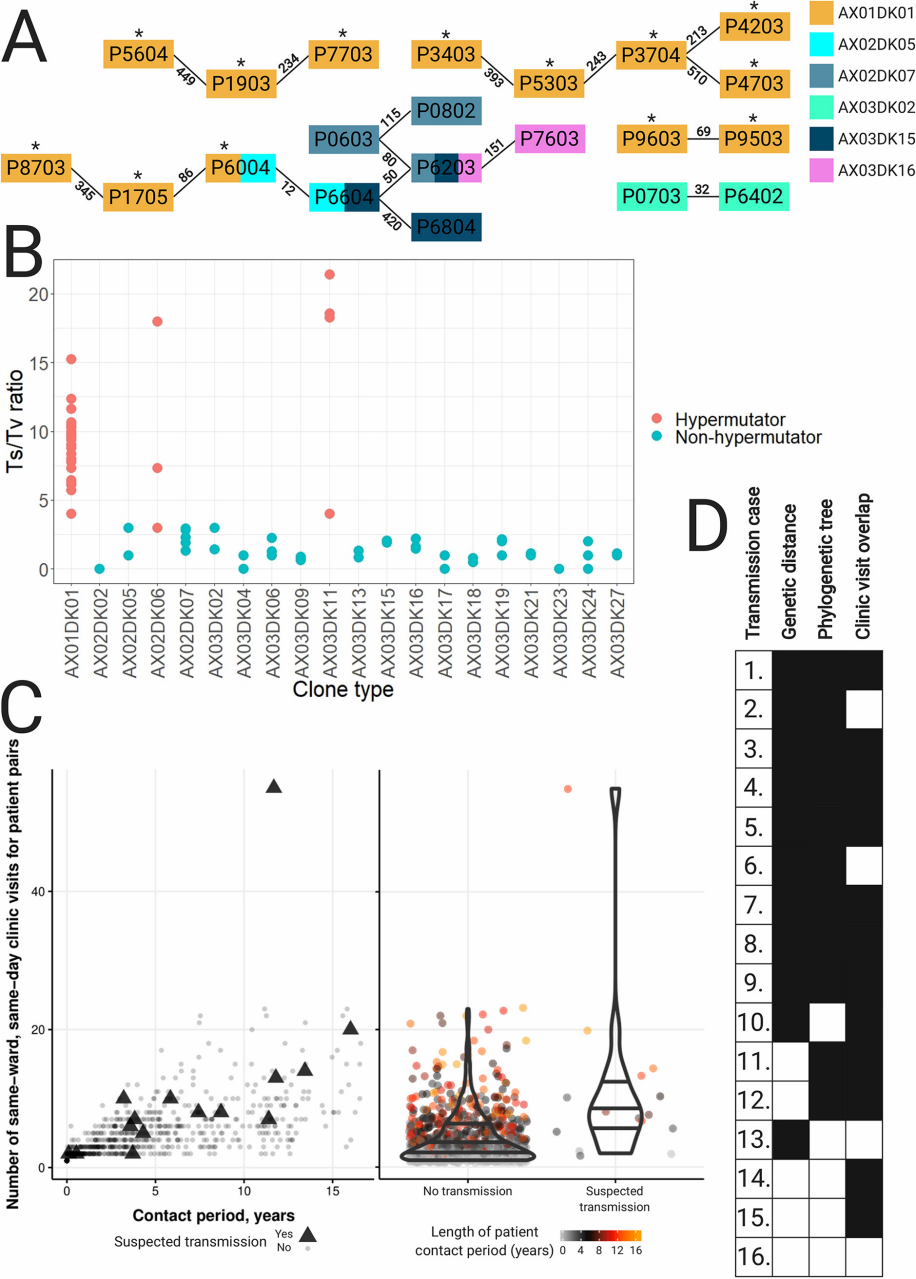
Mutational and transmission analysis of *Achromobacter* isolates. (A) All suspected patient-to-patient transmission cases identified. The smallest pairwise SNV distances between the isolates are given above the lines. Hypermutators are marked with asterisks. AX01, A. ruhlandii isolates; AX02, A. insuavis isolates; AX03, A. xylosoxidans isolates. (B) Transition-to-transversion substitution ratios for 20 *Achromobacter* clone types. (C) (Left) Number of times a patient pair visited the same hospital ward on the same date versus the time from first to last potential contact (in years). Patients with suspected transmission are marked with triangles. (Right) Distribution of patient contacts (by number of same-ward, same-day clinic visits) for patient pairs with suspected transmission versus the rest of the patient pair cohort. The color of each dot corresponds to the length of the patient contact period in years (from the first to the last contact date of the patient pair). (D) Summary of genetic, phylogenetic, and clinic visit overlap support for each suspected transmission case. Instances where cases have support for suspected transmission are shown in black.

Six of the clone types were found in more than one patient (range, 2 to 13 patients); thus, we were interested in finding out whether sharing of clone types was due to transmission between patients. We identified clonal isolate pairs that represented minimal SNV distances between isolates from different patients, and we defined these pairs as 16 suspected transmission cases for further investigation (Table 1; Fig. 3A).

**TABLE 1:** Smallest SNV and gene content distances within and between lineages involved in patient-to-patient transmission^*a*^

Case	Patient 1	Patient 2	Clone type	SNV distance			Gene distance		
				Between patients	Within patient 1	Within patient 2	Between patients	Within patient 1	Within patient 2
1	P9603	P9503	AX01DK01	69–368	182–323	117–275	11–115	94–124	9–15
2	P1705	P6004	AX01DK01	86–201	193–310		19–42	33–39	
3	P3704	P4203	AX01DK01	213–883	201–297		43–133	121–162	
4	P1903	P7703	AX01DK01	234–347	341	38	25–69	54	45
5	P5303	P3704	AX01DK01	243–672	346–553	201–297	41–185	4–54	121–162
6	P8703	P1705	AX01DK01	345–533		193–310	22–53		33–39
7	P3403	P5303	AX01DK01	393–469	653	346–553	16–71	35	4–54
8	P5604	P1903	AX01DK01	449–1,060	594	341	17–77	32	54
9	P3704	P4703	AX01DK01	510–631	201–297		41–137	121–162	
10	P6004	P6604	AX02DK05	12			6		
11	P0603	P6203	AX02DK07	80–93	29		372–411	77	
12	P0603	P0802	AX02DK07	115–184	29	92	410–614	77	16
13	P0703	P6402	AX03DK02	32			198		
14	P6203	P6604	AX03DK15	50			307		
15	P6604	P6804	AX03DK15	390			886		
16	P6203	P7603	AX03DK16	151–174		9–32	392–401		16–25

a SNV distance is the number of SNVs that are different between samples; gene distance is the number of genes that are different between samples.

In 12 suspected transmission cases (cases 1 to 9, 11, 12, and 16), we had multiple clonal isolates from at least one of the paired patients, enabling us to compare within-patient and between-patient genetic diversity (SNV and gene distances). In 9 of the 12 cases, we found that isolates from different patients were more closely genetically related than isolates from the same patient (cases 1 to 9 in Table 1). For example, AX01DK01 isolates from sibling patients P9603 and P9503 differed by 69 SNVs and 11 genes, whereas within-patient diversity was 117 to 323 SNVs and 9 to 124 genes (Table 1).

Next, we generated phylogenetic trees to reconstruct the genetic relationships of all four suspected patient-to-patient transmitted clone types for which three or more isolates were available—AX01DK01 (A. ruhlandii), AX02DK07 (A. insuavis), AX03DK15 (A. xylosoxidans), and AX03DK16 (A. xylosoxidans)—in order to find phylogenetic support for transmission events. Among 14 suspected transmission cases for which phylogenetic information was available, phylogenetic trees showed support for transmission in 11 (cases 1 to 9, 11, and 12 in Table 1), where isolates from one patient were phylogenetic descendants of isolates from another patient (Fig. S2).

We did not have enough isolates available for cases 10 and 13 to determine within-patient genetic diversity or phylogenies. Nonetheless, the clonal isolate pairs in the suspected cases showed only 12 and 32 SNV differences, respectively. As such, the SNV distances between isolates from patients in cases 10 and 13 were less than the within-patient genetic distances observed for any of the patients for whom we had multiple clonal isolates, except for AX02DK07 isolates from patient P0603, which showed 29 SNV differences. Accordingly, we found that the relatively short genetic distances between patient isolates supported the suspicion of transmission for cases 10 and 13.

To add to the genetic evidence of transmission, we analyzed the overlaps of patient visits to the clinic. We used our local CF database to count the number of days on which pairs of patients had microbial sampling in the same hospital ward (i.e., contact days). Patients were in potential contact with 8 to 45 other patients between 2002 and 2018. In total, we identified 3,522 patient contact days distributed across 804 patient pairs (out of a possible 1,275 patient pairs). Of the 16 patient pairs with suspected transmission events, only 1 patient pair (P8703 and P1705) never had microbial sampling in the same hospital ward on the same day. When we analyzed all patients with at least one contact day, we found that suspected transmissions tended to happen in patients with more contact days than nontransmission patients (median patient contacts, 8 versus 3, respectively; *P*, 2.7 × 10^−4^ by the Wilcoxon rank sum test) (Fig. 3C). Clinic visit data further support the suspected transmission in 12 cases (cases 1, 3 to 5, 7 to 12, 14, and 15 [see Table S5 for more information]) where potential between-patient contact happened before the first isolation of the transmitted clone type. Overall, 15 of 16 suspected between-patient transmission cases were supported by genetic distance, phylogenetic data, and/or epidemiological data (Fig. 3D). Only suspected patient-to-patient transmission between P6203 and P7603 had no supporting evidence.

A. ruhlandii clone type AX01DK01 showed the most suspected transmissions and was represented by 27 isolates across 13 patients. Therefore, we compared the genomes of AX01DK01 isolates to 19 genomes of clone type DES (A. ruhlandii), which has previously been reported to be frequently transmitted among Danish CF patients (10). We included the genomes of DES isolates from both the Copenhagen CF Center (*n* = 12) and the Aarhus CF Center (*n* = 7), and our analysis confirmed that clone types AX01DK01 and DES are the same (Fig. S1).

### Hypermutators are found only in chronic infections.

In a previous report, clone type AX01DK01/DES was shown to be hypermutable, putatively due to a 36-nucleotide (nt) in-frame deletion in the DNA mismatch repair (MMR) gene *mutS* (10). We found the same *mutS* deletion in all AX01DK01/DES isolates from this study, and the isolates showed large genetic diversity driven by an excess of transition substitutions (Fig. 3B) (e.g., isolates from patient P3403 were different by 677 SNVs, of which 624 were transitions), which is consistent with hypermutation caused by a defective DNA MMR system.

Next, we tested if hypermutation was evident in the other 19 clone types for which two or more isolates were available. We found that AX02DK06 and AX03DK11 of the species A. insuavis and A. xylosoxidans, respectively, also showed excess numbers of transition substitutions; thus, we also defined these two clone types as hypermutable. We also searched for mutations in the DNA MMR genes *mutS* and *mutL* in these two clone types, but we did not find that the mutations identified were always associated with an excess of transition substitutions. Finally, we noted that hypermutable clone types were found exclusively in patients clinically defined as chronically infected (Fig. 1).

### Development of antibiotic resistance over time.

We were able to retrieve routine clinical diagnostic measurements of susceptibility profiles against 21 antibiotics for all 92 isolates sampled from 2002 onward. For the 21 patients for whom only single isolates were available, the isolates were resistant or intermediately resistant to a median of 14 antibiotics. For the 30 patients for whom we included longitudinally collected isolates, we found early and late isolates to be resistant or intermediately resistant to a median of 14 and 18 antibiotics, respectively. The Wilcoxon rank sum test showed that late isolates were statistically significantly less susceptible than early (*P* = 3.9 × 10^−3^) and single (*P* = 5.0 × 10^−4^) isolates. Nearly all (87 to 92) isolates were resistant or intermediately resistant to the following nine antibiotics: ATM, CRO, CXM, CIP, MXF, PEN, RIF, TOB, and TMP. In contrast, no antibiotic was effective against all isolates, but many (51 to 63) isolates were susceptible to the following five antibiotics: IPM, MEM, TZP, SMZ, and SXT (Fig. 2B; also Table S3). Clone type AX01DK01/DES isolates were resistant or intermediately resistant to a median of 20 antibiotics, whereas for other *Achromobacter* isolates, the median number of such antibiotics was 14. The Wilcoxon rank sum test showed a significant difference between the two groups (*P* = 2.3 × 10^−7^). No statistically significant difference between the median numbers of resistant or intermediately resistant A. insuavis and A. xylosoxidans isolates was identified (*P* = 0.92). Interestingly, 7 of 29 A. ruhlandii isolates (6 of 27 AX01DK01/DES isolates) were resistant or intermediately resistant to all 21 antibiotics, while only 1 A. insuavis isolate and none of the A. xylosoxidans isolates were resistant or intermediately resistant to all antibiotics.

## DISCUSSION

*Achromobacter* is an emerging pathogen causing chronic respiratory tract infections in patients with CF; however, the genetic epidemiology of these infections is not well understood. We sequenced and analyzed 101 genomes of *Achromobacter* isolates from 51 patients with CF. This is the largest longitudinally collected *Achromobacter* genome data set available to date.

Our analysis revealed that nearly 20% of patients were infected with two to four *Achromobacter* species and/or clone types over the sampling period, suggesting that not all *Achromobacter* strains colonizing the airways lead to chronic infections and further supporting the early antibiotic treatment of *Achromobacter* infections (9). Besides, the frequent observation of multiple *Achromobacter* species and/or clone types in the same patient emphasizes the necessity for a sensitive species- and clone type-level typing scheme for *Achromobacter* in order to distinguish infections caused by different *Achromobacter* species and/or different *Achromobacter* clone types. Phylogenetic analysis showed that MALDI-TOF or API 20 typing is not accurate for *Achromobacter* species-level typing, as has also been recently indicated by others (31–33); thus, we suggest that sequencing of marker genes (e.g., *bla*_OXA_ or *nrdA*) or WGS should be used for species-level typing in the clinical setup (34). WGS, furthermore, can facilitate patient-to-patient transmission identification.

The majority of *Achromobacter* infections are acquired from the environment, and the prevalence of patient-to-patient transmission remains controversial: while some studies have identified patients infected with unique clone types of *Achromobacter* (35–37), other studies have reported cases of suspected patient-to-patient transmission (7, 30, 38, 39). We suspected cases of transmission between patients for all three *Achromobacter* species in our study. Of 16 suspected transmission cases, 15 were supported by genetic distance measurements, phylogenetic trees, and/or epidemiological data. Transmission between patients P1705 and P6004 was defined as an indirect transmission event by Hansen et al. in 2013 (30). P9603 and P9503 are siblings who share the same living environment; therefore, transmission of CF airway pathogen between them is highly likely. Between-patient transmission of A. insuavis has not been reported in the scientific literature before.

We note that the observed SNV distances between the transmitted *Achromobacter* isolates are higher than those observed for transmitted P. aeruginosa (40); this complicates the identification of transmission and might explain why no patient-to-patient transmission was discovered in several previous studies (35–37). We showed, furthermore, that gene content differences between isolates could serve as an additional criterion for between-patient transmission identification.

Interestingly, A. ruhlandii AX01DK01/DES—a known hypermutator—is widely transmitted between patients with CF within and between CF centers in Copenhagen and Aarhus, even though some studies show reduced transmissibility of hypermutator strains (41). We showed that isolates did not group phylogenetically according to center origin, suggesting multiple transmission events between centers. This phenomenon might be explained by patients with CF physically moving from one city and CF center to another. Furthermore, recent first-time isolation of AX01DK01/DES in two patients with CF (P7703 and P8703) indicates that while transmission of AX01DK01/DES has been recognized previously (10), additional actions might need to be taken to prevent such transmission.

Finally, we observed that infection of several patients with the same clone type was generally a sign of suspected patient-to-patient transmission of *Achromobacter*. This knowledge could be applied in diagnostics, where sharing of an *Achromobacter* clone type would be the first sign of suspected transmission between patients and would lead to further investigations.

Moreover, we confirmed previous findings that A. ruhlandii clone type AX01DK01/DES is hypermutable, and in addition, we found evidence of hypermutation in A. insuavis and A. xylosoxidans clone types (10). Hypermutation could be pivotal for *Achromobacter* persistence, since all hypermutable clone types caused chronic infections in patients with CF.

We confirmed that *Achromobacter* is highly antibiotic resistant and, further, that late isolates from patients colonized with *Achromobacter* were significantly more antibiotic resistant than early or single isolates. This indicates that *Achromobacter* adapts to the host environment where high levels of antibiotics are present, even though *Achromobacter* is innately highly antibiotic resistant. Moreover, the significantly higher antibiotic resistance of AX01DK01/DES than of other *Achromobacter* isolates calls for additional efforts to prevent AX01DK01/DES transmission between patients with CF.

Our study has several limitations. First, while our data set spans several decades, the number of isolates for each patient is low, and we have selected for isolates to represent patients with longitudinal samples, leading to overrepresentation of chronically infected patients. More sequenced isolates would have allowed us to more accurately identify suspected transmission events and to better follow hypermutability and antibiotic resistance development. Furthermore, we used single isolates to represent a heterogeneous *Achromobacter* population in patients with CF, which could have led to a lack of evidence in some suspected between-patient transmissions. Finally, we did not know if patients had met outside the hospital or during microbial sampling, and we used only potential contact information, which adds to the uncertainty about patient contacts. Nevertheless, our study provides evidence for between-patient transmission in all three *Achromobacter* species, the development of hypermutability caused by deletions in the *mutS* gene, and the development of antibiotic resistance over time.

### Conclusions.

In summary, by sequencing the genomes of the largest data set of *Achromobacter* clinical isolates from patients with CF to date, we confirmed that the MALDI-TOF and API N20 methods are unsuitable for *Achromobacter* species-level typing, and we conclude that WGS is the most appropriate method for species-level typing and patient-to-patient transmission identification. Moreover, we confirmed that sequencing of only one isolate is not sufficient, since multiple patients were infected with different *Achromobacter* species or clone types. For the first time, we identified suspected between-patient transmission of A. insuavis. Furthermore, we found genomic and epidemiological support for suspected patient-to-patient transmission in all three *Achromobacter* species, and we suggest a new measure—gene content difference—to be taken into account in evaluating suspected between-patient transmission cases. We also emphasized that a shared clone type is the first sign of possible patient-to-patient transmission. Finally, we showed that antibiotic resistance develops in all three *Achromobacter* species, and our analysis confirmed previous findings that hypermutability is associated with chronic *Achromobacter* infections (10, 12). The results of this work allow us to better understand antibiotic resistance dynamics and patient-to-patient transmission of *Achromobacter* in patients with CF, which could help predict the clinical progression of *Achromobacter* infections and prevent patient-to-patient transmission.

## Supplementary Material

Supplemental file 1
