# Supplementary material for: Transmission and Antibiotic Resistance of Achromobacter in Cystic Fibrosis
Source: J Clin Microbiol. 2021 Mar 19;59(4):e02911-20. doi: 10.1128/JCM.02911-20 (PMC8092725; doi:10.1128/JCM.02911-20)
Supplement: Supplemental file 1 [file JCM.02911-20-s0001.pdf]

Supplementary information

Table S1. List of all patients attending Copenhagen CF Center who were infected with *Achromobacter* in 2002–2018.

Patient ID	Chronic	Number of isolates taken	Number of isolates sequenced	Date of the first isolate*	Species as defined by MALDI-TOF/API N20
P1700	No	2	1	2013	A. xylosoxidans
P4600	No	10	0	2010	A. xylosoxidans
P3901	Yes	127	0	2002	A. xylosoxidans
P4501	No	8	0	2002	A. xylosoxidans/A. insolitus
P7201	Yes	68	0	2009	A. xylosoxidans
P0802	Yes	120	2	2002	A. xylosoxidans
P1102	No	1	0	2005	A. xylosoxidans
P2202	No	1	1	2011	A. xylosoxidans
P2802	No	5	0	2002	A. denitrificans
P3602	No	4	0	2002	A. xylosoxidans/A. denitrificans
P5602	Yes	100	0	2002	A. xylosoxidans
P5802	No	5	0	2004	A. xylosoxidans
P6402	Yes	5	1	2017	A. xylosoxidans
P6602	Yes	134	0	2007	A. xylosoxidans
P7402	No	4	1	2014	A. xylosoxidans
P9902	Yes	137	3	2002	A. xylosoxidans
P0603	No	24	2	2002	A. xylosoxidans
P0703	Yes	18	2	2011	A. xylosoxidans
P0803	Yes	58	3	2002	A. xylosoxidans
P0903	Yes	111	1	2002	A. xylosoxidans/A. denitrificans
P1703	Yes	40	1	2010	A. xylosoxidans
P1903	Yes	166	4	2002	A. xylosoxidans
P2103	No	3	1	2016	A. xylosoxidans
P3203	Yes	238	4	2002	A. xylosoxidans
P3403	Yes	113	2	2002	A. xylosoxidans
P3503	Yes	79	0	2002	A. xylosoxidans
P4003	Yes	119	0	2002	A. xylosoxidans
P4203	Yes	49	2	2012	A. xylosoxidans
P4303	No	4	0	2010	A. denitrificans
P4703	Yes	157	3	2002	A. xylosoxidans
P4903	No	3	0	2002	A. denitrificans
P5103	No	7	1	2015	A. xylosoxidans
P5303	Yes	221	3	2003	A. xylosoxidans
P5803	Yes	46	1	2011	A. xylosoxidans
P5903	No	3	0	2003	A. xylosoxidans
P6203	No	4	4	2006	A. xylosoxidans
P6703	No	2	0	2012	A. xylosoxidans

P6803	No	6	0	2002	A. xylosoxidans
P7003	Yes	147	0	2002	A. xylosoxidans
P7403	Yes	74	0	2008	A. xylosoxidans
P7603	Yes	98	4	2008	A. xylosoxidans
P7703	Yes	9	2	2017	A. xylosoxidans
P8003	Yes	52	0	2004	A. xylosoxidans
P8103	No	2	0	2004	A. xylosoxidans
P8603	No	30	2	2002	A. xylosoxidans
P8703	Yes	60	1	2003	A. denitrificans
P8903	No	1	0	2005	A. xylosoxidans
P9203	Yes	38	3	2011	A. xylosoxidans
P9403	Yes	125	3	2004	A. xylosoxidans
P9503	Yes	93	3	2007	A. xylosoxidans
P9603	Yes	128	3	2003	A. xylosoxidans
P9803	No	1	0	2010	A. denitrificans
P0004	No	2	0	2014	A. xylosoxidans
P0104	No	9	0	2006	A. xylosoxidans
P0604	No	3	1	2003	A. xylosoxidans
P0904	Yes	53	0	2009	A. xylosoxidans
P1004	Yes	147	0	2009	A. xylosoxidans
P1304	Yes	7	0	2002	A. xylosoxidans
P1504	Yes	32	0	2008	A. xylosoxidans
P1704	Yes	9	2	2002	A. xylosoxidans
P1804	No	2	0	2002	A. xylosoxidans
P2104	No	1	0	2011	A. xylosoxidans
P3004	Yes	3	1	2007	A. xylosoxidans/A. insolitus
P3104	No	5	0	2008	A. xylosoxidans
P3504	No	8	2	2013	A. xylosoxidans
P3704	Yes	175	3	2003	A. xylosoxidans
P4104	No	3	1	2014	A. xylosoxidans
P4704	No	5	0	2011	A. xylosoxidans
P4904	Yes	23	0	2008	A. xylosoxidans
P5204	No	4	1	2007	A. xylosoxidans
P5604	Yes	48	2	2007	A. xylosoxidans
P6004	Yes	80	2	2007	A. xylosoxidans
P6104	No	3	0	2004	A. xylosoxidans/A. denitrificans
P6604	Yes	64	2	2007	A. xylosoxidans
P6804	No	2	1	2006	A. xylosoxidans
P7204	No	3	0	2006	A. xylosoxidans
P8004	No	8	0	2010	A. xylosoxidans/A. denitrificans/A. insolitus
P8104	No	1	1	2011	A. xylosoxidans
P8204	No	12	0	2003	A. xylosoxidans
P8404	Yes	61	3	2013	A. xylosoxidans
P8504	No	6	2	2014	Achromobacter/A. insolitus
P8704	No	1	0	2010	A. xylosoxidans

P9604	No	5	0	2005	A. xylosoxidans
P9704	Yes	86	2	2010	A. xylosoxidans/A. denitrificans
P0105	No	1	0	2005	A. xylosoxidans
P0305	No	1	0	2006	A. denitrificans
P1005	No	2	0	2011	A. xylosoxidans
P1305	Yes	14	0	2002	A. xylosoxidans
P1605	No	2	0	2011	A. insolitus
P1705	Yes	111	3	2007	A. xylosoxidans
P2005	No	4	1	2017	A. xylosoxidans
P2605	No	4	1	2016	A. xylosoxidans/A. insolitus
P6005	No	3	0	2014	A. xylosoxidans
P6905	No	3	0	2014	A. xylosoxidans
P8605	No	8	2	2013	A. xylosoxidans
P0610	Yes	71	2	2011	A. xylosoxidans
P5611	No	1	1	2017	A. xylosoxidans
P8511	Yes	10	1	2017	A. xylosoxidans
P3205	No	2	0	2016	A. xylosoxidans
P1512	No	19	0	2016	A. xylosoxidans
P2206	No	1	0	2018	A. xylosoxidans
P2905	No	2	0	2015	A. xylosoxidans
P8705	No	1	0	2018	A. xylosoxidans
P3103	No	4	0	2018	A. xylosoxidans

* In some cases where the first isolate date is 2002, it is the first documented isolate and not the first isolate taken from the patient (there are no records of isolates taken prior to 2002)

Table S2. Information about isolate sequencing, alignment and assembly quality.

Isolate	Number of reads	Percent of positions with Q-score≥30 in R1	Percent of positions with Q-score≥30 in R2	Average alignment coverage	Percent of the reference genome positions considered in the analysis (coverage in all samples >=10x)	Number of scaffolds in assemblies	Scaffold N50
P1700_04112013_AX03DK01-0	1236967	91.26	66.71	44.9702	NA	160	184708
P0802_04042005_AX02DK07-0	1921055	70.96	52.25	53.1081	74.78	202	146329
P0802_18012008_AX02DK07-0	1473581	73.63	44.65	40.5247	74.78	153	158164
P2202_04012011_AX04DK01-0	1064406	76.75	28.58	24.2448	NA	190	332669
P6402_27032017_AX03DK02-0	679888	77.38	42.16	25.1953	83.41	224	73783
P7402_26022014_AX01DK02-0	1203047	88.87	74.3	61.8133	NA	191	134541
P9902_01011996_AX02DK06-0	1231124	89.39	73.4	53.5014	71.80	199	149072
P9902_01032004_AX02DK06-0	350677	80.32	57.8	15.5908	71.80	257	44696
P9902_26012009_AX02DK06-0	1062546	89.84	73.41	46.3418	71.80	148	146618
P0603_03012007_AX02DK07-0	1231059	81.22	60.89	39.8374	74.78	216	102583
P0603_08102003_AX02DK07-0	1313583	83.18	57.2	43.6965	74.78	235	131247
P0703_07082013_AX03DK02-0	1460584	89.01	75.85	63.1056	83.41	394	141080
P0703_23092011_AX03DK05-0	1046818	88.99	72.38	47.5802	NA	299	107564

P0803_01012000_AX03DK06-0	1508023	91.06	74.17	52.6904	86.20	117	132136
P0803_26042014_AX03DK06-0	1146138	90.71	67.3	37.7869	86.20	381	97234
P0803_27042007_AX03DK06-0	1280237	90.69	71.51	43.9816	86.20	165	141101
P0903_06012012_AX03DK07-0	886554	86.69	70.53	38.8576	NA	149	122906
P1703_16032010_AX03DK08-0	994577	71.9	27.64	33.2292	NA	231	99435
P1903_01011996_AX03DK09-0	865492	89.15	77.79	36.574	86.34	192	104382
P1903_01092016_AX01DK01-0	960654	89.87	75.55	45.4156	80.85	319	85559
P1903_16122011_AX01DK01-0	1006102	90.2	74.36	47.0012	80.85	191	79969
P1903_27032006_AX03DK09-0	997956	89.52	73.05	41.5704	86.34	158	124447
P2103_11042016_AX03DK10-0	694537	93.89	81.78	31.5362	NA	171	125703
P3203_01011997_AX03DK11-0	1655094	88.06	75.6	74.9065	85.20	185	128643
P3203_07022014_AX03DK11-0	755267	83.8	59.6	34.0323	85.20	171	75846
P3203_18042005_AX03DK11-0	1660733	87.62	76.14	74.6272	85.20	180	146696
P3203_19092012_AX03DK11-0	1611854	87.28	73.2	74.6853	85.20	191	129689
P3403_06102003_AX01DK01-0	1311320	91.58	73.46	52.5772	80.85	172	106229
P3403_27022012_AX01DK01-0	1445803	91.44	74.36	56.2188	80.85	191	92936
P4203_09102012_AX03DK12-0	1234301	91.23	72.05	47.1957	NA	204	133994
P4203_09122014_AX01DK01-0	1184740	90.54	72.61	48.9751	80.85	197	85654
P4703_01012000_AX03DK13-0	1365747	92.09	79.39	48.844	73.83	252	115850
P4703_16022007_AX03DK13-0	1253012	92.28	78.16	44.8413	73.83	281	129622
P4703_18022015_AX01DK01-0	1183348	90.65	80.26	56.9179	80.85	202	85301
P5103_07102016_AX02DK09-0	1584763	89.27	72.24	70.8556	NA	137	131939
P5303_07102016_AX01DK01-0	919446	89.84	75.36	42.588	80.85	173	85489
P5303_14012004_AX01DK01-0	982584	89.99	73.89	45.3862	80.85	315	70232
P5303_14102009_AX01DK01-0	955166	88.99	78.27	45.1233	80.85	183	92674
P5803_17012011_AX03DK14-0	919088	86.25	73.1	41.9749	NA	126	142009
P6203_01012000_AX03DK15-0	1298566	90.18	74.85	52.0941	79.32	295	116827
P6203_02102006_AX03DK16-0	1114110	88.3	64.66	47.1393	81.83	232	192141
P6203_12122015_AX02DK08-0	765735	91.86	83.36	36.3228	NA	283	73708
P6203_24092018_AX02DK07-0	663419	91.3	84.03	24.2781	74.78	203	82444
P7603_01011997_AX04DK02-0	1284557	89.53	80.92	39.6519	NA	92	282949
P7603_04042014_AX03DK16-0	1343593	81.1	56.88	55.7502	81.83	150	139100
P7603_18022011_AX03DK16-0	615534	77.71	57.14	26.5182	81.83	241	86498
P7603_19112008_AX03DK16-0	803956	77.41	64.51	34.1174	81.83	134	112119
P7703_16032018_AX01DK01-0	1177983	88.37	77.21	55.3489	80.85	363	86775
P7703_19012017_AX01DK01-0	1217025	88.12	72.22	53.8748	80.85	244	88018
P8603_03072008_AX03DK17-0	1403388	72.45	44.68	48.9187	85.67	322	168641
P8603_27082014_AX03DK17-0	1423853	73.85	46.54	49.8143	85.67	183	162600
P8703_15032017_AX01DK01-0	1338572	77.62	52.07	50.5233	80.85	256	85370
P9203_01012000_AX02DK03-0	976984	90.55	78.17	44.4988	NA	225	124753
P9203_08042016_AX03DK18-0	1179899	91.93	79.26	53.8671	84.40	146	167726
P9203_13122011_AX03DK18-0	763636	86.69	76.25	34.4134	84.40	172	102487
P9403_05092016_AX03DK19-0	1068025	91.47	79.46	47.3566	86.36	163	119797
P9403_29092004_AX03DK19-0	1126849	91.38	79.21	51.1977	86.36	210	153581
P9403_30102009_AX03DK19-0	1205610	92.18	77.46	52.8502	86.36	174	134733
P9503_02012007_AX01DK01-0	1393880	85.72	76.54	68.2448	80.85	416	103435

P9503_04032011_AX01DK01-0	796367	87.06	71.93	40.0553	80.85	384	73675
P9503_27052014_AX01DK01-0	1637852	88.01	76.13	77.5176	80.85	189	100034
P9603_01042014_AX01DK01-0	1140742	88.54	76.12	55.1843	80.85	266	74400
P9603_28102003_AX01DK01-0	1508188	87.41	72.37	74.6452	80.85	166	114598
P9603_28112008_AX01DK01-0	1568086	88.98	76.26	73.1531	80.85	246	106324
P0604_30122003_AX03DK20-0	1708579	89.73	73.37	74.2587	NA	276	186108
P1704_01011999_AX03DK21-0	439827	74.88	17.6	16.1498	68.86	370	61153
P1704_07022011_AX03DK21-0	2118817	73.61	26.55	61.7486	68.86	132	137960
P3004_24042007_AX03DK22-0	1523025	85.33	65.92	52.6253	NA	117	162189
P3504_09012015_AX03DK23-0	1659379	83.64	60.97	65.7022	87.97	174	140329
P3504_20122013_AX03DK23-0	1511020	84.4	58.7	58.9443	87.97	172	119989
P3704_08022008_AX01DK01-0	1196741	90.43	74.28	54.9169	80.85	506	84063
P3704_26112003_AX01DK01-0	1357889	90.41	71.6	62.6729	80.85	358	89603
P3704_27062014_AX01DK01-0	1377576	90.69	73.11	61.8987	80.85	234	126894
P4104_02122014_AX01DK03-0	1337224	91.62	68.6	53.63	NA	137	124338
P5204_15082016_AX03DK03-0	814201	82.8	81.13	31.1042	NA	280	104233
P5604_07102016_AX01DK01-0	1155400	90.13	80.88	56.5172	80.85	185	79929
P5604_28082007_AX01DK01-0	1242997	91.95	76.33	58.8504	80.85	248	114652
P6004_19062007_AX01DK01-0	891956	88.43	64.55	40.7879	80.85	198	71531
P6004_30102015_AX02DK05-0	1056268	91.8	76.31	47.6295	80.78	156	138080
P6604_17022016_AX02DK05-0	459481	87.19	65.5	19.407	80.78	178	84561
P6604_25012007_AX03DK15-0	493397	87.69	70.78	19.7344	79.32	223	78447
P6804_08032006_AX03DK15-0	1018439	89.75	75.94	42.9768	79.32	304	108018
P8104_29042011_AX02DK10-0	1266695	90.32	62.94	45.2698	NA	185	101894
P8404_11122013_AX03DK24-0	683978	68.4	54.39	26.925	83.38	175	114190
P8404_11122013_AX03DK24-1	1303761	83.88	63.1	47.9031	83.38	148	132577
P8404_13022014_AX03DK24-0	1307025	82.15	64.8	50.6423	83.38	120	117081
P8504_05012016_AX02DK02-0	941910	89.11	71.38	43.5086	89.14	222	121959
P8504_05012016_AX02DK02-1	736226	89.53	64.43	34.5314	89.14	152	98857
P9704_12082016_AX03DK25-0	876563	92.2	81.77	39.1596	NA	123	147550
P9704_29072010_AX02DK01-0	997031	90.89	76.85	44.9791	NA	195	91010
P1705_21052013_AX01DK01-0	1278632	80.07	60.35	57.4422	80.85	241	88276
P1705_25012010_AX01DK01-0	626179	80.01	64.99	28.742	80.85	280	61198
P1705_30032007_AX01DK01-0	467709	84.53	59.21	21.3413	80.85	240	52853
P2005_03022017_AX02DK04-0	989926	72.47	48.05	38.126	NA	364	69143
P2605_06072016_AX03DK26-0	654352	93.53	79.73	32.3912	NA	128	140329
P8605_01062016_AX03DK04-0	1235358	90.7	74.76	56.1584	85.59	127	125201
P8605_07052018_AX03DK04-0	746743	89.08	57.38	31.3722	85.59	244	97497
P0610_14092016_AX03DK27-0	900398	89.84	73.11	40.775	86.23	159	106137
P0610_26012012_AX03DK27-0	987659	89.52	73.67	43.6624	86.23	145	107606
P5611_13012017_AX03DK28-0	1176229	89.23	71.68	45.3802	NA	296	142304
P8511_07042017_AX03DK29-0	586843	76.46	47.22	17.686	NA	229	84010

Table S3. Isolate susceptibility data to 21 tested antibiotics. R—resistant to the antibiotic, I—intermediately resistant to the antibiotic, S—susceptible to the antibiotic. 1-Amoxicillin/Clavulanate; 2-Ampicillin; 3-

Aztreonam; 4-Ceftazidime; 5-Ceftriaxone; 6-Cefuroxime;7-Chloramphenicol; 8-Ciprofloxacin; 9-Colistin; 10-Imipenem; 11-Meropenem; 12-Moxifloxacin; 13-Penicillin; 14- Piperacillin/Tazobactam; 15-Rifampicin; 16-Sulfonamide; 17-Tetracycline; 18-Tigecycline; 19- Tobramycin; 20-Trimethoprim; 21-Trimethoprim/sulfamethoxazole.

**Isolate number**	**Species**	**1**	**2**	**3**	**4**	**5**	**6**	**7**	**8**	**9**	**10**	**11**	**12**	**13**	**14**	**15**	**16**	**17**	**18**	**19**	**20**	**21**
P1700-04112013-AX03DK01-0	*A. xylosoxidans*	S	I	R	I	R	R	S	R	S	S	S	R	R	S	R	S	R	R	R	R	S
P0802-04042005-AX02DK07-0	*A. insuavis*	R	R	R	R	R	R	I	R	R	S	R	R	R	S	NA	S	S	NA	R	R	S
P0802-18012008-AX02DK07-0	*A. insuavis*	I	R	R	R	R	R	S	R	R	S	S	R	R	I	R	S	S	I	R	R	S
P2202-04012011-AX04DK01-0	*New genotype*	I	S	R	S	I	R	S	I	S	S	S	I	R	S	I	S	S	S	R	R	S
P6402-27032017-AX03DK02-0	*A. xylosoxidans*	R	R	R	I	R	R	R	I	I	S	S	R	R	S	R	S	R	R	R	R	S
P7402-26022014-AX01DK02-0	*A. ruhlandii*	R	R	R	I	R	R	R	R	I	R	R	R	R	I	R	R	R	R	R	R	R
P9902-01032004-AX02DK06-0	*A. insuavis*	R	R	R	I	R	R	S	R	S	S	I	R	R	S	NA	R	R	NA	R	R	I
P9902-26012009-AX02DK06-0	*A. insuavis*	R	R	R	R	R	R	R	R	I	S	R	R	R	S	R	R	R	R	R	R	R
P0603-08102003-AX02DK07-0	*A. insuavis*	S	R	R	R	R	R	I	R	S	S	S	R	R	S	NA	S	S	NA	R	R	S
P0603-03012007-AX02DK07-0	*A. insuavis*	S	R	R	S	S	I	NA	I	I	S	S	I	R	S	NA	S	S	NA	R	R	S
P0703-23092011-AX03DK05-0	*A. xylosoxidans*	S	R	R	S	R	R	R	R	I	I	S	R	R	S	R	S	R	I	R	R	S
P0703-07082013-AX03DK02-0	*A. xylosoxidans*	S	I	R	I	R	R	S	I	I	S	S	R	R	S	R	S	R	R	R	R	S
P0803-27042007-AX03DK06-0	*A. xylosoxidans*	I	R	R	I	R	R	NA	I	R	S	NA	R	R	S	NA	S	R	NA	R	R	S
P0803-26042014-AX03DK06-0	*A. xylosoxidans*	S	R	R	I	R	R	S	R	R	S	S	R	R	S	R	S	R	R	R	R	S
P0903-06012012-AX03DK07-0	*A. xylosoxidans*	I	I	R	I	R	R	R	R	S	S	S	R	R	S	R	R	R	R	R	R	R
P1703-16032010-AX03DK08-0	*A. xylosoxidans*	I	S	R	S	R	R	I	I	S	S	S	R	R	S	R	S	R	R	R	R	S
P1903-27032006-AX03DK09-0	*A. xylosoxidans*	I	R	R	R	R	R	NA	I	R	R	I	R	R	S	NA	S	R	NA	R	R	S
P1903-16122011-AX01DK01-0	*A. ruhlandii*	R	R	R	R	R	R	R	R	R	I	R	R	R	R	R	R	R	S	I	R	R

P1903-01092016-AX01DK01-0	*A. ruhlandii*	R	R	R	R	R	R	R	R	R	I	R	R	R	R	R	R	R	S	R	R	R
P2103-11042016-AX03DK10-0	*A. xylosoxidans*	S	R	R	I	R	R	R	I	I	S	S	R	R	S	R	S	R	S	R	R	S
P3203-07022014-AX03DK11-0	*A. xylosoxidans*	R	R	R	R	R	R	R	R	R	I	R	R	R	R	R	S	R	R	R	R	R
P3203-18042005-AX03DK11-0	*A. xylosoxidans*	NA	R	R	S	R	R	R	R	S	S	S	R	R	S	NA	R	R	NA	R	R	I
P3203-19092012-AX03DK11-0	*A. xylosoxidans*	R	R	R	R	R	R	R	R	S	S	R	R	R	S	R	R	R	S	R	R	R
P3403-06102003-AX01DK01-0	*A. ruhlandii*	R	R	R	R	R	R	R	R	R	R	R	R	R	S	NA	I	R	NA	R	R	R
P3403-27022012-AX01DK01-0	*A. ruhlandii*	R	R	R	S	R	R	S	R	S	I	R	R	R	S	I	R	S	S	R	R	I
P4203-09102012-AX03DK12-0	*A. xylosoxidans*	S	I	R	I	R	R	I	R	S	S	S	R	R	S	R	S	R	S	R	R	S
P4203-09122014-AX01DK01-0	*A. ruhlandii*	R	R	R	R	R	R	S	R	R	R	R	R	R	I	R	S	R	S	R	R	S
P4703-16022007-AX03DK13-0	*A. xylosoxidans*	S	R	R	S	R	R	NA	R	I	S	NA	R	R	S	NA	S	R	NA	R	R	S
P4703-18022015-AX01DK01-0	*A. ruhlandii*	R	R	R	R	R	R	R	R	R	R	R	R	R	R	R	R	R	S	R	R	R
P5103-07102016-AX02DK09-0	*A. insuavis*	R	R	R	I	R	R	R	R	S	S	S	R	R	S	R	S	R	R	R	R	S
P5303-14012004-AX01DK01-0	*A. ruhlandii*	S	R	R	S	R	R	R	R	R	S	S	R	R	S	NA	S	R	NA	R	R	S
P5303-14102009-AX01DK01-0	*A. ruhlandii*	R	R	R	R	R	R	R	R	R	R	R	R	R	R	R	R	R	R	R	R	R
P5303-07102016-AX01DK01-0	*A. ruhlandii*	R	R	R	R	R	R	R	R	R	R	R	R	R	I	R	R	R	S	R	R	R
P5803-17012011-AX03DK14-0	*A. xylosoxidans*	I	R	R	S	R	R	R	R	I	S	S	R	R	S	R	S	R	S	R	R	S
P6203-02102006-AX03DK16-0	*A. xylosoxidans*	I	R	R	S	R	R	NA	I	I	I	S	R	R	S	NA	S	R	NA	R	R	S
P6203-12122015-AX02DK08-0	*A. insuavis*	R	R	R	I	R	R	S	I	S	S	S	R	R	S	R	S	S	S	R	R	S
P6203-24092018-AX02DK07-0	*A. insuavis*	S	R	R	R	R	R	R	R	S	NA	S	NA	R	S	R	S	S	S	R	R	S
P7603-19112008-AX03DK16-0	*A. xylosoxidans*	S	R	R	S	R	R	S	I	S	R	S	R	R	S	I	S	R	R	R	R	S

P7603-18022011-AX03DK16-0	*A. xylosoxidans*	I	R	R	I	R	R	I	I	I	I	S	R	R	S	R	S	R	S	R	R	S
P7603-04042014-AX03DK16-0	*A. xylosoxidans*	R	R	R	R	R	R	S	R	R	R	R	R	R	I	R	S	R	R	R	R	S
P7703-19012017-AX01DK01-0	*A. ruhlandii*	R	R	R	R	R	R	R	R	R	I	R	R	R	R	R	R	R	S	R	R	R
P7703-16032018-AX01DK01-0	*A. ruhlandii*	R	R	R	R	R	R	S	R	R	NA	R	NA	R	I	R	R	R	S	R	R	R
P8603-27082014-AX03DK17-0	*A. xylosoxidans*	S	R	R	I	R	R	S	R	I	S	S	R	R	S	R	S	R	S	R	R	S
P8603-03072008-AX03DK17-0	*A. xylosoxidans*	I	R	R	S	R	R	I	R	R	S	S	R	R	S	R	S	R	I	R	R	S
P8703-15032017-AX01DK01-0	*A. ruhlandii*	R	R	R	R	NA	R	R	R	R	S	I	R	R	S	R	R	R	S	R	R	R
P9203-13122011-AX03DK18-0	*A. xylosoxidans*	I	I	R	S	R	R	I	I	I	S	S	R	R	S	R	S	R	S	R	R	S
P9203-08042016-AX03DK18-0	*A. xylosoxidans*	R	R	R	R	R	R	R	R	I	S	S	R	R	S	R	S	R	R	R	R	S
P9403-29092004-AX03DK19-0	*A. xylosoxidans*	S	I	R	S	R	R	R	R	I	S	S	R	R	S	NA	S	R	NA	R	R	S
P9403-30102009-AX03DK19-0	*A. xylosoxidans*	I	R	R	S	R	R	I	I	S	S	S	R	R	S	R	S	R	S	R	R	S
P9403-05092016-AX03DK19-0	*A. xylosoxidans*	R	R	R	R	R	R	R	R	R	S	I	R	R	I	R	S	R	S	R	R	S
P9503-02012007-AX01DK01-0	*A. ruhlandii*	R	R	R	I	R	R	NA	R	R	R	R	R	R	R	NA	S	R	NA	R	R	S
P9503-04032011-AX01DK01-0	*A. ruhlandii*	R	R	R	R	R	R	R	R	R	R	R	R	R	R	R	I	R	R	R	R	S
P9503-27052014-AX01DK01-0	*A. ruhlandii*	R	R	R	R	R	R	R	R	R	R	R	R	R	R	R	R	R	R	R	R	R
P9603-28102003-AX01DK01-0	*A. ruhlandii*	S	R	R	R	R	R	R	R	S	S	S	R	R	R	NA	S	R	NA	R	R	S
P9603-28112008-AX01DK01-0	*A. ruhlandii*	R	R	R	R	R	R	R	R	R	R	R	R	R	I	R	S	R	R	R	R	S
P9603-01042014-AX01DK01-0	*A. ruhlandii*	R	R	R	R	R	R	R	R	R	R	R	R	R	I	R	R	R	R	R	R	R
P0604-30122003-AX03DK20-0	*A. xylosoxidans*	S	S	R	S	R	R	I	R	S	S	S	R	R	S	NA	S	R	NA	R	R	S
P1704-07022011-AX03DK21-0	*A. xylosoxidans*	R	R	R	I	R	R	I	R	R	S	S	R	R	S	R	S	R	R	S	R	S

P3004-24042007-AX03DK22-0	*A. xylosoxidans*	S	I	R	S	R	R	NA	I	I	S	NA	R	R	S	NA	S	R	NA	R	R	S
P3504-20122013-AX03DK23-0	*A. xylosoxidans*	S	I	R	I	R	R	R	R	I	S	S	R	R	S	R	S	R	S	R	R	S
P3504-09012015-AX03DK23-0	*A. xylosoxidans*	S	I	R	I	R	R	R	R	R	S	S	R	R	S	R	S	R	S	R	R	S
P3704-26112003-AX01DK01-0	*A. ruhlandii*	S	R	R	R	R	R	R	R	R	S	S	R	R	S	NA	R	R	NA	R	R	I
P3704-08022008-AX01DK01-0	*A. ruhlandii*	R	R	R	R	R	R	R	R	R	R	R	R	R	R	R	R	R	R	R	R	R
P3704-27062014-AX01DK01-0	*A. ruhlandii*	R	R	R	R	R	R	R	R	R	R	R	R	R	R	R	R	R	R	R	R	R
P4104-02122014-AX01DK03-0	*A. ruhlandii*	S	R	R	I	R	R	R	S	S	S	S	R	R	S	R	S	R	R	R	R	S
P5204-15082016-AX03DK03-0	*A. xylosoxidans*	S	R	R	I	R	R	R	I	I	S	S	R	R	S	R	S	R	S	R	R	S
P5604-28082007-AX01DK01-0	*A. ruhlandii*	S	R	R	R	R	R	NA	R	R	S	S	R	R	S	NA	R	R	NA	R	R	R
P5604-07102016-AX01DK01-0	*A. ruhlandii*	R	R	R	R	R	R	R	R	R	R	R	R	R	R	R	R	R	S	R	R	R
P6004-19062007-AX01DK01-0	*A. ruhlandii*	R	R	R	R	R	R	NA	R	R	R	R	R	R	R	NA	S	R	NA	R	R	S
P6004-30102015-AX02DK05-0	*A. insuavis*	R	R	R	R	R	R	R	R	R	R	R	R	R	R	R	R	R	R	R	R	R
P6604-25012007-AX03DK15-0	*A. xylosoxidans*	I	R	R	S	R	R	NA	R	S	S	NA	R	R	S	NA	S	R	NA	R	R	S
P6604-17022016-AX02DK05-0	*A. insuavis*	I	R	R	R	R	R	R	I	S	S	S	R	R	S	R	S	S	S	R	R	S
P6804-08032006-AX03DK15-0	*A. xylosoxidans*	I	R	R	S	R	R	NA	R	I	S	S	R	R	S	NA	S	R	NA	R	R	S
P8104-29042011-AX02DK10-0	*A. insuavis*	I	R	R	I	R	R	R	I	S	S	S	R	R	S	R	S	S	R	R	I	S
P8404-11122013-AX03DK24-0	*A. xylosoxidans*	S	I	R	R	R	R	R	R	I	S	S	R	R	S	R	S	R	R	R	R	S
P8404-11122013-AX03DK24-1	*A. xylosoxidans*	S	I	R	R	R	R	R	R	I	S	S	R	R	S	R	S	R	R	R	R	S
P8404-13022014-AX03DK24-0	*A. xylosoxidans*	S	S	R	R	R	R	R	R	I	S	I	R	R	S	R	S	R	R	R	R	S
P8504-05012016-AX02DK02-0	*A. insuavis*	R	R	R	R	R	R	R	I	S	S	S	R	R	S	R	S	R	R	R	R	S

P8504-05012016-AX02DK02-1	*A. insuavis*	R	R	R	R	R	R	R	I	S	S	S	R	R	S	R	S	R	R	R	R	S
P9704-12082016-AX03DK25-0	*A. xylosoxidans*	I	I	R	S	R	R	R	R	R	S	S	R	R	S	R	S	R	S	R	R	S
P9704-29072010-AX02DK01-1	*A. insuavis*	NA	NA	R	I	NA	NA	NA	I	S	S	S	R	NA	S	NA	NA	NA	NA	R	NA	NA
P1705-30032007-AX01DK01-0	*A. ruhlandii*	R	R	R	R	R	R	NA	R	R	R	NA	R	R	R	NA	R	R	NA	R	R	I
P1705-25012010-AX01DK01-0	*A. ruhlandii*	R	R	R	R	R	R	R	R	R	R	R	R	R	S	R	R	R	R	R	R	R
P1705-21052013-AX01DK01-0	*A. ruhlandii*	R	R	R	R	R	R	R	R	R	R	R	R	R	I	R	R	R	S	R	R	R
P2005-03022017-AX02DK04-0	*A. insuavis*	R	R	R	R	R	R	R	R	S	I	R	R	R	R	R	R	R	S	R	R	R
P2605-06072016-AX03DK26-0	*A. xylosoxidans*	S	R	R	I	R	R	R	R	I	S	S	R	R	S	R	S	R	R	R	R	S
P8605-01062016-AX03DK04-0	*A. xylosoxidans*	R	R	R	I	R	R	R	R	S	S	S	R	R	S	R	S	R	S	R	R	S
P8605-07052018-AX03DK04-0	*A. xylosoxidans*	I	R	R	I	R	R	R	R	S	NA	S	NA	R	S	R	S	R	S	R	R	S
P0610-26012012-AX03DK27-0	*A. xylosoxidans*	R	R	R	I	R	R	R	R	R	S	I	R	R	I	R	S	R	R	R	R	S
P0610-14092016-AX03DK27-0	*A. xylosoxidans*	R	R	R	R	R	R	R	R	R	S	R	R	R	R	R	R	R	S	R	R	R
P5611-13012017-AX03DK28-0	*A. xylosoxidans*	S	R	R	I	R	R	R	R	I	S	S	R	R	S	R	S	R	S	R	R	S
P8511-07042017-AX03DK29-0	*A. xylosoxidans*	I	R	R	I	R	R	S	I	S	S	S	R	R	S	R	S	R	S	R	R	S

Table S4. MLST profiles of 101 *Achromobacter* isolates.

**Sample**	**Clone type**	**ST**
P1903_01092016_AX01DK01-0	AX01DK01	385
P1903_16122011_AX01DK01-0	AX01DK01	385
P3403_06102003_AX01DK01-0	AX01DK01	385
P3403_27022012_AX01DK01-0	AX01DK01	-
P4203_09122014_AX01DK01-0	AX01DK01	385
P4703_18022015_AX01DK01-0	AX01DK01	385
P5303_07102016_AX01DK01-0	AX01DK01	385
P5303_14012004_AX01DK01-0	AX01DK01	-
P5303_14102009_AX01DK01-0	AX01DK01	385

P7703_16032018_AX01DK01-0	AX01DK01	385
P7703_19012017_AX01DK01-0	AX01DK01	385
P8703_15032017_AX01DK01-0	AX01DK01	385
P9503_02012007_AX01DK01-0	AX01DK01	385
P9503_04032011_AX01DK01-0	AX01DK01	385
P9503_27052014_AX01DK01-0	AX01DK01	385
P9603_01042014_AX01DK01-0	AX01DK01	385
P9603_28102003_AX01DK01-0	AX01DK01	385
P9603_28112008_AX01DK01-0	AX01DK01	385
P3704_08022008_AX01DK01-0	AX01DK01	385
P3704_26112003_AX01DK01-0	AX01DK01	385
P3704_27062014_AX01DK01-0	AX01DK01	385
P5604_07102016_AX01DK01-0	AX01DK01	-
P5604_28082007_AX01DK01-0	AX01DK01	385
P6004_19062007_AX01DK01-0	AX01DK01	385
P1705_21052013_AX01DK01-0	AX01DK01	-
P1705_25012010_AX01DK01-0	AX01DK01	385
P1705_30032007_AX01DK01-0	AX01DK01	-
P7402_26022014_AX01DK02-0	AX01DK02	-
P4104_02122014_AX01DK03-0	AX01DK03	-
P9704_29072010_AX02DK01-0	AX02DK01	-
P8504_05012016_AX02DK02-0	AX02DK02	-
P8504_05012016_AX02DK02-1	AX02DK02	-
P9203_01012000_AX02DK03-0	AX02DK03	-
P2005_03022017_AX02DK04-0	AX02DK04	-
P6004_30102015_AX02DK05-0	AX02DK05	-
P6604_17022016_AX02DK05-0	AX02DK05	-
P9902_01011996_AX02DK06-0	AX02DK06	-
P9902_01032004_AX02DK06-0	AX02DK06	-
P9902_26012009_AX02DK06-0	AX02DK06	-
P0802_04042005_AX02DK07-0	AX02DK07	144
P0802_18012008_AX02DK07-0	AX02DK07	144
P0603_03012007_AX02DK07-0	AX02DK07	144
P0603_08102003_AX02DK07-0	AX02DK07	144
P6203_24092018_AX02DK07-0	AX02DK07	144
P6203_12122015_AX02DK08-0	AX02DK08	-
P5103_07102016_AX02DK09-0	AX02DK09	-
P8104_29042011_AX02DK10-0	AX02DK10	218
P1700_04112013_AX03DK01-0	AX03DK01	5
P6402_27032017_AX03DK02-0	AX03DK02	11
P0703_07082013_AX03DK02-0	AX03DK02	11
P5204_15082016_AX03DK03-0	AX03DK03	-
P8605_01062016_AX03DK04-0	AX03DK04	-
P8605_07052018_AX03DK04-0	AX03DK04	-
P0703_23092011_AX03DK05-0	AX03DK05	-
P0803_01012000_AX03DK06-0	AX03DK06	326

P0803_26042014_AX03DK06-0	AX03DK06	326
P0803_27042007_AX03DK06-0	AX03DK06	326
P0903_06012012_AX03DK07-0	AX03DK07	201
P1703_16032010_AX03DK08-0	AX03DK08	-
P1903_01011996_AX03DK09-0	AX03DK09	426
P1903_27032006_AX03DK09-0	AX03DK09	426
P2103_11042016_AX03DK10-0	AX03DK10	184
P3203_01011997_AX03DK11-0	AX03DK11	228
P3203_07022014_AX03DK11-0	AX03DK11	228
P3203_18042005_AX03DK11-0	AX03DK11	-
P3203_19092012_AX03DK11-0	AX03DK11	228
P4203_09102012_AX03DK12-0	AX03DK12	-
P4703_01012000_AX03DK13-0	AX03DK13	-
P4703_16022007_AX03DK13-0	AX03DK13	-
P5803_17012011_AX03DK14-0	AX03DK14	387
P6203_01012000_AX03DK15-0	AX03DK15	27
P6604_25012007_AX03DK15-0	AX03DK15	27
P6804_08032006_AX03DK15-0	AX03DK15	-
P6203_02102006_AX03DK16-0	AX03DK16	182
P7603_04042014_AX03DK16-0	AX03DK16	182
P7603_18022011_AX03DK16-0	AX03DK16	182
P7603_19112008_AX03DK16-0	AX03DK16	182
P8603_03072008_AX03DK17-0	AX03DK17	184
P8603_27082014_AX03DK17-0	AX03DK17	-
P9203_08042016_AX03DK18-0	AX03DK18	-
P9203_13122011_AX03DK18-0	AX03DK18	-
P9403_05092016_AX03DK19-0	AX03DK19	237
P9403_29092004_AX03DK19-0	AX03DK19	237
P9403_30102009_AX03DK19-0	AX03DK19	237
P0604_30122003_AX03DK20-0	AX03DK20	-
P1704_01011999_AX03DK21-0	AX03DK21	200
P1704_07022011_AX03DK21-0	AX03DK21	200
P3004_24042007_AX03DK22-0	AX03DK22	-
P3504_09012015_AX03DK23-0	AX03DK23	282
P3504_20122013_AX03DK23-0	AX03DK23	282
P8404_11122013_AX03DK24-0	AX03DK24	175
P8404_11122013_AX03DK24-1	AX03DK24	175
P8404_13022014_AX03DK24-0	AX03DK24	175
P9704_12082016_AX03DK25-0	AX03DK25	153
P2605_06072016_AX03DK26-0	AX03DK26	-
P0610_14092016_AX03DK27-0	AX03DK27	-
P0610_26012012_AX03DK27-0	AX03DK27	-
P5611_13012017_AX03DK28-0	AX03DK28	-
P8511_07042017_AX03DK29-0	AX03DK29	-
P2202_04012011_AX04DK01-0	AX04DK01	-
P7603_01011997_AX04DK02-0	AX04DK02	-

Table S5. Suspected transmission cases with clinic visitation overlap information and comments whether the epidemiological data supports the transmission.

Patient 1	Patient 2	Clone type	Total contact dates	Time period	Date of first contact	Date of last contact	First isolate of the transmitted clone type		Support for transmission
							Patient 1	Patient 2	
P1903	P7703	AX01DK01	2	0.5	2015.9	2016.4	2016.3	2017.1	Yes, exposure before known colonization of Patient 2
P6402	P0703	AX03DK02	2	0.1	2018.6	2018.7	2017.2	2011.7	No, contact occurs after colonization
P6203	P7603	AX03DK16	2	3.7	2011.2	2014.9	2006.8	2008.9	No, contact occurs after colonization
P4203	P3704	AX01DK01	6	3.5	2013.4	2016.9	2014.9	2003.9	Yes, exposure before known colonization of Patient 1
P6203	P6604	AX03DK15	7	11.4	2005.9	2017.3	2000.0	2007.1	Yes, exposure before known colonization of Patient 2
P0802	P0603	AX02DK07	7	3.8	2003.9	2007.7	2005.3	2003.8	Yes, exposure before known colonization of Patient 1
P4703	P3704	AX01DK01	8	8.7	2003.7	2012.4	2015.1	2003.9	Yes, exposure before known colonization of Patient 1
P6004	P1705	AX01DK01	10	3.2	2007.5	2010.7	2007.5	2007.2	No, patient contact on the day of clone type detection
P6604	P6804	AX03DK15	10	5.8	2006.8	2012.6	2007.1	2006.2	Yes, patient contact before colonization of Patient 1
P0603	P6203	AX02DK07	14	13.4	2005.4	2018.8	2003.8	2018.7	Yes, exposure before known colonization of Patient 2
P6004	P6604	AX02DK05	13	11.8	2005.9	2017.7	2015.8	2016.1	Yes, exposure before known colonization of Patient 2
PP5303	P3704	AX01DK01	20	16	2002.9	2018.9	2004.0	2003.9	Yes, exposure before known colonization of Patient 1
P9503	P9603	AX01DK01	55	11.7	2005	2016.7	2007.0	2003.8	Yes, exposure before known colonization of Patient 1. Patients are siblings, thus transmission in the household has likely occurred.
P8703	P1705	AX01DK01	0	-	-	-	2017.2	2007.2	No, no contact between patients
P3403	PP5303	AX01DK01	8	7.4	2002.1	2009.5	2003.8	2004.0	Yes, patient contact before colonization of Patient 2
P5604	P1903	AX01DK01	5	4.4	2005.8	2010.2	2007.7	2016.3	Yes, patient contact before colonization of Patient 2

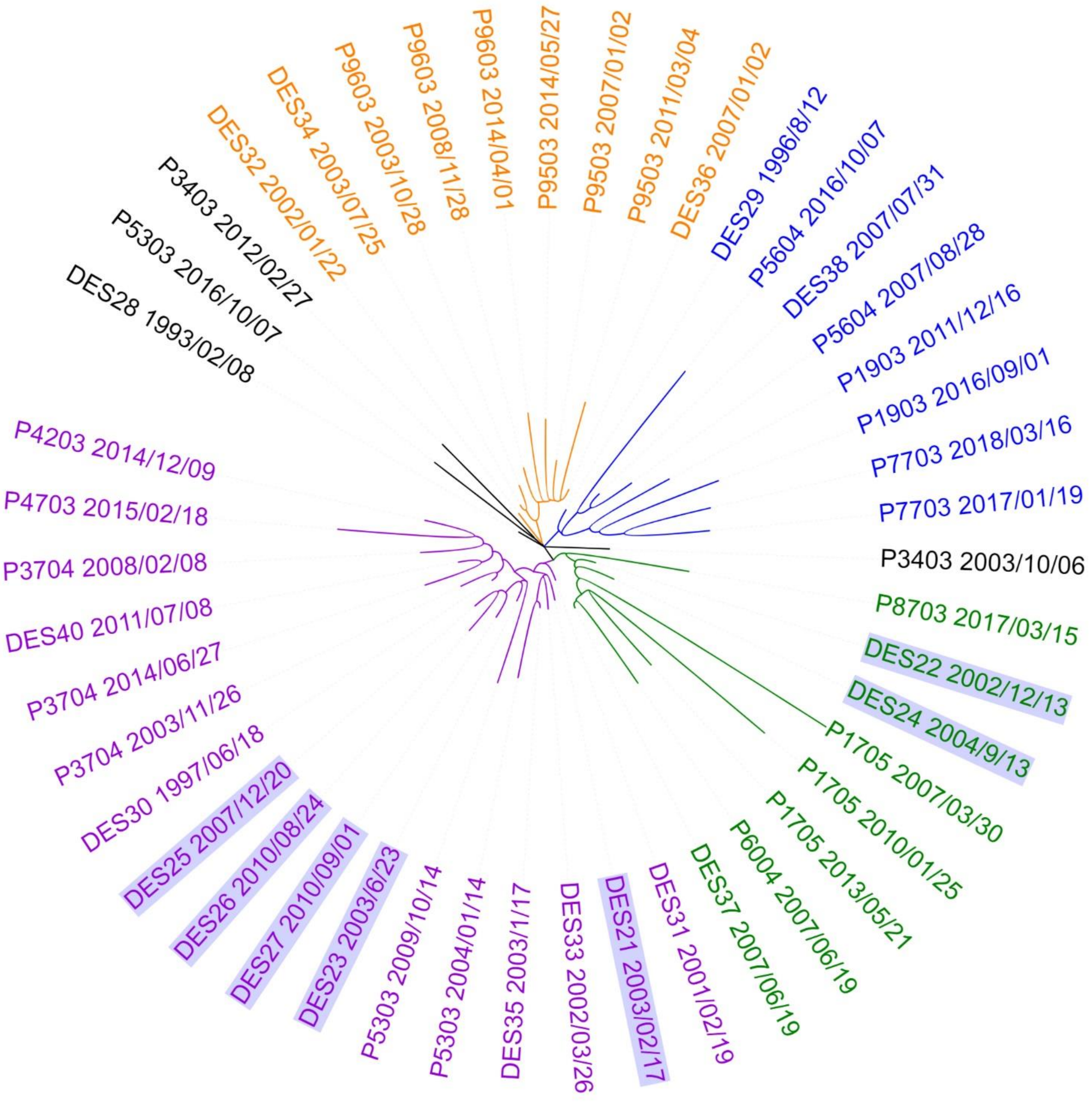

Figure S1. Core genome SNV-based phylogenetic tree from *de novo* assembled DES isolates of which 7 were from patients attending CF center in Aarhus (blue label background; (10)) and 25 were from patients attending CF center in Copenhagen. The four patient clusters are separated by branch and label colors. The phylogenetic tree can be accessed on iTOL webserver: https://itol.embl.de/tree/12807362379081584977362.

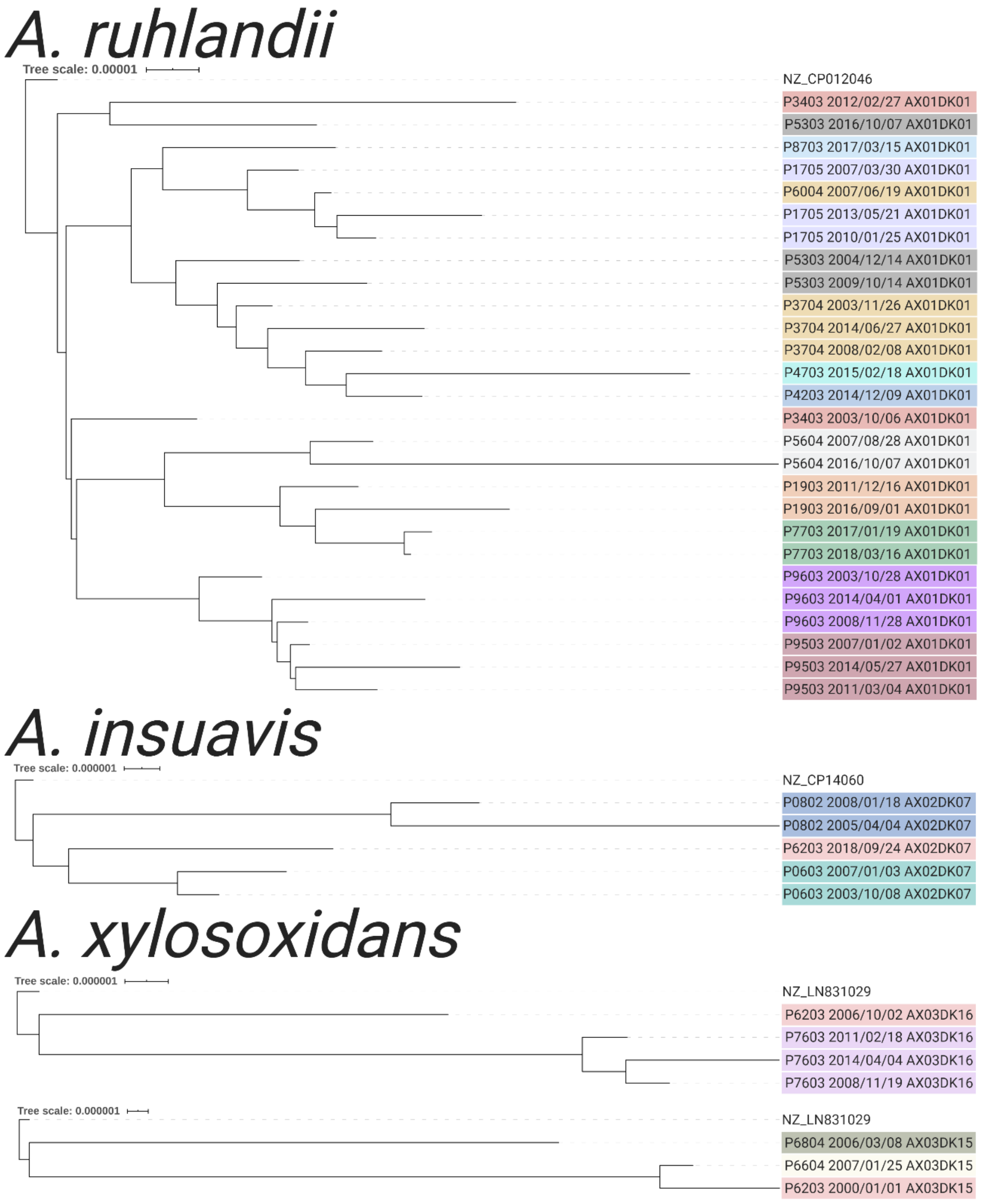

Figure S2. Core genome SNV phylogenetic trees of suspected bacterial isolates transmission between patients in *Achromobacter ruhlandii*, *A. insuavis*, and *A. xylosoxidans* where 4 or more isolates were available. Isolates are named as follows: [Patient ID] [Sampling date] [Species and clone type].
